# Evolution of the ASF Infection Stage in Wild Boar Within the EU (2014–2018)

**DOI:** 10.3389/fvets.2020.00155

**Published:** 2020-04-01

**Authors:** Marta Martínez-Avilés, Irene Iglesias, Ana De La Torre

**Affiliations:** Centro de Investigación en Sanidad Animal (CISA), INIA, Valdeolmos, Spain

**Keywords:** antibodies, epidemiology, surveillance, moderately virulent virus, survivor, African swine fever, wild boar

## Abstract

African swine fever (ASF) is one of the most important emerging transboundary diseases of pigs, causing trade restrictions, and a health impact on susceptible pigs. Nine countries in the continental European Union (Estonia, Lithuania, Latvia, Poland, Czech Republic, Bulgaria, Belgium, Romania, and Hungary) have been affected by ASF from 2014 to 2018 and it keeps spreading despite the efforts to control it. For a number of years, we have witnessed high case-fatality rates in wild boar found dead particularly in new infected areas, which is typical of the peracute and acute forms of the infection at the beginning of an ASF epidemic. Experimental evidence with currently circulating strains indicates that some infected animals can remain asymptomatic and might even survive the infection. An increased presence of virus of moderate virulence can complicate ASF diagnosis as well as the mitigation and control of the disease. We analyze the ASF surveillance data in wild boar in the four EU countries where ASF has been present for longer, comparing the spatial density of antibody positive notifications with the time ASF has been present per region. Results indicate an increasing annual distribution of notifications based on antibodies over nucleic acid detection in hunted wild boar in Estonia, Latvia and Poland. Potentially, Lithuania, and Poland seem to have experienced more acute forms in 2017 and 2018 than Latvia and Estonia. Overall there was a positive statistical correlation between time with infection (TWI) and antibody positive density, with some variations in certain regions, particularly of Lithuania and Estonia. The increasing trend in potential survivors (hunted wild boar with confirmed PCR negative and antibody positive results) enhances the importance of surveillance design to sample and test shot wild boar. In conclusion, surveillance data based on ASFV detection by PCR and serology can be used to assess the status of the epidemic in wild boar.

## Introduction

Wild boar in Estonia, Latvia, Lithuania, and Poland have been affected by African swine fever (ASF) since 2014, following the spread from other Eastern European countries where the disease had been expanding since its first occurrence in this part of the world in 2007. ASF continued spreading within the European Union (EU), affecting the Czech Republic and Romania in 2017, Belgium, Bulgaria, and Hungary in 2018, and reaching the backyard pig population of Serbia and Slovakia in 2019 (1, 2). Despite the surveillance and control actions taken in the EU, ASF has continued to spread. ASF has since 2018 also quickly expanded in up to 10 countries in Asia including China, causing severe consequences within the pig industry. Of the 24 known genotypes of ASF virus (ASFV), only two have caused epidemics outside Africa: genotype 1 (1960–1990's, affecting mainly Spain and Portugal in Europe and reaching some countries in Central and South America) and genotype 2 (current epidemic in Europe and Asia).

Attempts to control the infection in wild boar in the current epidemic have only been successful in the Czech Republic (3). Wild boar is a challenge for ASF control since it is difficult to detect the infection early. The EU surveillance strategy in wild boar from 2015 and until its next review in 2021 is mainly based on the promotion of passive surveillance and active patrolling to find dead wild boar, with ASFV detection being the test of choice in the four epidemiological scenarios identified: free areas, free areas bordering infected areas, infected areas to control, infected areas to eradicate (ASF Strategy for the EU, SANTE/7113/2015-Rev 11). Antibody testing is recommended additionally for shot animals (sometimes referred to as culled and others as hunted) in the infected-to-control and infected-to-eradicate scenarios. The detection of antibodies is always indicative of infection since there is yet no safe commercial vaccine available (4) and should be used for the diagnosis of subacute and subclinical forms of ASF.

Moderately virulent ASFV are already currently circulating (5–8). These virulent viruses produce clinical signs and lesions that are compatible with the simultaneous occurrence of acute, subacute, and chronic forms of the disease. The incubation period is therefore variable and when it is longer than in acute infections, virus shedding is prolonged over time too, particularly since the percentage of animals that could survive the infection can oscillate between 50 and 75% of the population (6). The existence of survivor animals has been described in the current epidemic (8–10) but their role in ASFV spread is still under discussion within the scientific community.

It has been hypothesized that under stressing conditions, like hunting, drought, lack of food or concomitant infections, survivors that have apparently cleared the infection (negative to virus detection but antibody positive) can become infectious again (11). A prolonged shedding together with a higher percentage of survivors may therefore constitute a prolonged source of infection for other susceptible animals.

During the 1960–1980's, in the previous ASF epidemic outside Africa (with virus genotype 1), the disease was first detected in Portugal and subsequently in Spain. In <5 years since its introduction, increased numbers of subacute and chronic forms appeared (12). These modified forms spread insidiously and remained extremely difficult to diagnose. As a consequence, low and moderately virulent ASFV spread through the Iberian Peninsula and were introduced to other countries in Europe and Latin America, mostly through meat or meat products from pigs in which the infection was unnoticed and to which susceptible pigs were exposed to Mebus (13). The fall of pork prices in affected territories due to the restrictive control measures also contributed to spread ASF to neighboring countries (14). At the time, there seemed to be a higher awareness about the risk of ASFV spread through moderately pathogenic strains, since even in the presence of unspecific or contradictory clinical signs with a low mortality rate, samples were tested against ASF. The early laboratory confirmation together with hard but effective control measures like quick stamping out of the affected farm and all of its contacts, banning of transport and movements, and repopulation with sentinel animals previously quarantined, was sufficient to eradicate ASF in mainland Italy, France, Belgium, the Netherlands, and Cuba (15, 16). In the islands of Malta, Dominican Republic and Haiti, eradication was achieved when the whole swine population was destructed, but in Haiti the implementation of measures took longer and was a threat for other countries in the area (15, 17, 18). In Brazil, despite an early detection, ASF perpetuated through swill feeding, the presence of classical swine fever, and social factors that resulted in mistrust toward the situation of ASF in the country complicated control, that was finally achieved with the support of government and military police, the destruction in slaughterhouses of animals confirmed positive by the National Reference Laboratory with direct immunofluorescence and heamadsorption in leucocyte cultures for virus detection and indirect immunofluorescence and immunoelectrophoresis for antibody detection (19). Only Portugal, Spain and Sardinia remained endemic. The development of a sensitive and specific ELISA test in 1979 in Spain was one of the most important pillars to detect and eradicate ASF positive animals in an endemic situation. There was evidence of a small percentage (<5%) of survivors that were able to further transmit the virus, but their role in the maintenance of the disease in the population was not as frequent as other routes of transmission such as contacts among neighboring farms (17). Nonetheless, it was not until all survivors were eliminated, thus suppressing any possibility of any of them becoming carriers, that ASF was finally eradicated from the Iberian Peninsula in the 90's (20). A 2012 study also confirmed the absence of ASF in wild boar in the area that had been most affected (21).

Alternatively, other authors assume that survivors would not shed significant amounts of virus and would not represent a prolonged source of infection (10, 22, 23). These authors argue, among other reasons, that animals that survive ASF infection are rare in the current epidemiological situation. Schulz et al. (10) could only detect nine wild boar that were both ASF positive by PCR and serology by surveillance in an area in Estonia where seropositive animals dominate the epidemiological situation, possibly indicating the late phase of the epidemic. However, they recognize that ASF could become endemic instead of fading out. In any case, it is now clear that comprehensive surveillance and laboratory results based on ASFV detection by PCR and serology, can be used to assess the status of the epidemic in wild boar.

The aim of this study is to analyze the ASF surveillance data notified through the EU Animal Disease Notification System (ADNS) with the objective of characterizing the infection in wild boar in those areas in which ASF has been present for longer (Estonia, Latvia, Lithuania and Poland). Following ASF dynamics, one would expect to find a higher density of seropositive wild boar in those areas in which the infection has been present for longer.

## Materials and Methods

Each notification (confirmed ASF) in the ADNS database contains at least information on the host (wild boar/domestic pig), the location (latitude, longitude, region, and country), confirmation date, reference number, outbreak type (primary/secondary), and number of affected animals. There is space to add free text and countries generally include here other useful information in a non-systematic way: test results, found dead or hunted wild boar, age and gender, type of farm, location. We restricted the study to wild boar notifications.

From the free text, we were able to assign the category of dead/hunted for each wild boar notification. To do so, we searched for key terms like “hunted,” “shot,” “hunting,” “executed,” “killed,” “shoot,” to assign the “hunted” category, and “dead” or “found” for dead wild boar. The data were checked several times since, for example, some notifications included both the words hunted and dead in the text, and it was necessary to classify these on a one-by-one basis. When in the same notification there was information about both dead and hunted wild boar (*n* = 62), we favored the category “hunted” since our interest is primarily to analyze the evolution of infection when the disease might be unnoticed. However, if there was no information on whether the wild boar were either hunted or dead, we favored the category “dead” (*n* = 1,213).

Notifications were also classified according to whether the confirmation of infection had been performed by PCR, which we assumed represented the initial stages of infection (Stage 1); by PCR and an antibody test (ELISA and/or IPT), which we assumed would represent animals which had the infection for some time longer (Stage 2); or which were positive to the antibody test and the nucleic acid detection test was either not specified or negative, which we assume would represent the latest stage of infection, when ASFV detection decreases but immunity mounts, leading to an increased percentage of survivors (Stage 3). For 1,160 notifications (<10% of the total 12,661) with no information on whether the wild boar was hunted or found dead or on the test used, we assumed they were dead wild boar tested with PCR. Wild boar notifications estimated to be in Stage 3 of infection comprise those with a positive antibody result together with either those that specifically state that a negative PCR has been obtained or those in which we assume the PCR has been negative because this diagnostic test result is not specified.

Since only Estonia, Latvia, Lithuania, and Poland have confirmed the detection of antibody against ASF, we restricted the analysis of the evolution of ASF infection to these four countries. The temporal evolution of the notifications in dead and hunted wild boar and the diagnostic test/s specified in the notifications was analyzed descriptively for these four countries. Notifications comprised between 2014 and March 2019, but we restricted the analysis to complete years (2014–2018). Differences and trends were statistically analyzed in R Core Team (24).

For each administrative unit within a country, we estimated the time with infection (TWI) by subtracting the last from the first date in which ASF was notified to obtain the number of days ASF has been present in each unit. The assumption is that independently on whether the infection has remained or has been reintroduced, the probability of finding antibody will be higher the longer the infection has been present in that area (longer TWI). The administrative units used were “powiat” (second level, county or district) for Poland, “savivaldybe” (second level, municipality) for Lithuania, “aprinki|rajoni” (second level, district) for Latvia and “maakond” (first level, county) for Estonia, similar in size and publicly available for download at https://gadm.org (version 3.6, last accessed on June 2019). The estimated TWI was explored spatially by representing the distribution of natural breaks (Jenks) classification in a choropleth map in each administrative unit.

We explored whether there could be a correlation between the number of notifications in which antibodies were detected and the estimated TWI per administrative unit by computing Spearman's correlation coefficient Rho in R Core Team (24), where a *p* <0.05 was considered statistically significant. The same analysis was also carried out with the proportion of notifications in which antibodies were detected and TWI.

The ASF wild boar notifications with positive serology were fitted a kernel density function in a map, using geodesic distances between points and an output cell size of 0.034 sq km. Both maps were developed in ArcGIS 10.2 (ESRI) and were compared qualitatively.

## Results

**Figure 1:**
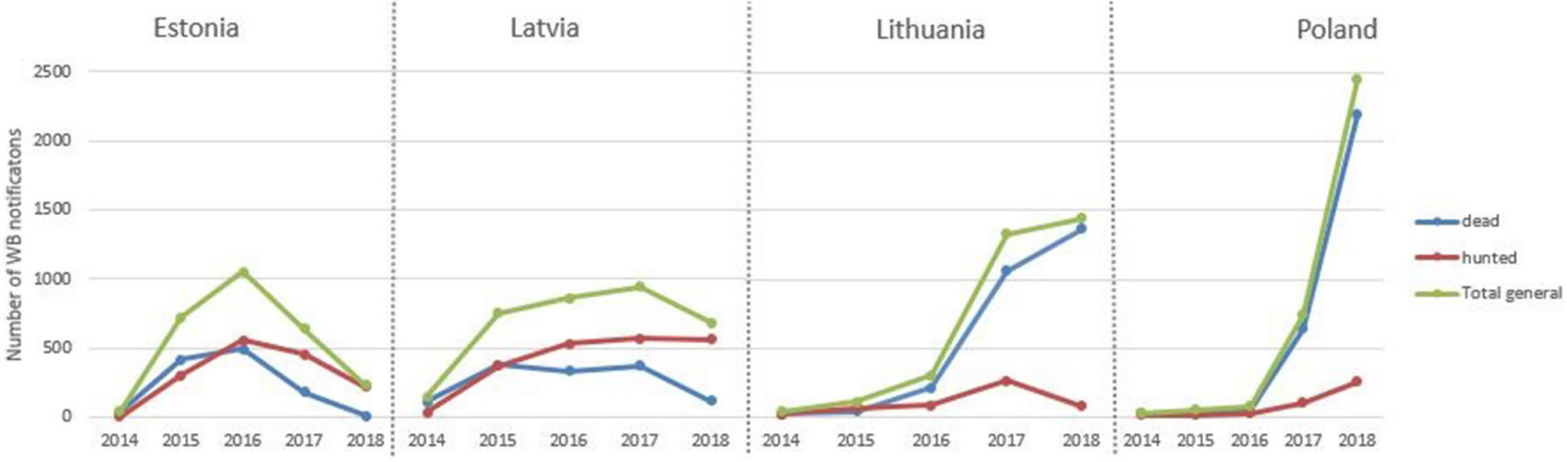
Annual number of ASF notifications in wild boar.

From the entry of ASF in Eastern EU in 2014 to December 2018 there have been 13,379 wild boar notifications to the ADNS from EU countries, of which 95% (12,661) have occurred in Estonia, Latvia, Lithuania and Poland, which have been the only countries in the EU infected since 2014. In these 4 countries, ASF has been detected in over 8,100 wild boar found dead (64%) and over 4,500 hunted (36%). The annual evolution of ASF positive wild boar found dead and hunted in Estonia, Latvia, Lithuania, and Poland from 2014–2018 is represented in Figure 1. Lithuania and Poland mainly notified ASF in wild boar from dead animals. Lithuania and Poland have increased the number of notifications in wild boar each year, while Estonia's notifications in wild boar peaked in 2016 and Latvia's in 2017.

The annual distribution of notifications by diagnostic test used and estimated stage of infection is shown in Table 1. The majority of notifications (78%, 9,882) were based on PCR results (Stage 1). The remaining 22% comprise 393 notifications that include both PCR and antibody positivity results (Stage 2) and 2,386 notifications based on antibody results only (Stage 3).

**Table 1:** Annual distribution of ASF notifications in wild boar by diagnostic test/s used and estimated stage of infection.

**Year**	**Stage 1**	**Stage 2**	**Stage 3**
	**(PCR+, AB-)^a^**	**(PCR+, AB+)^b^**	**(PCR–, AB+)^c^**
2014	243	19	2
2015	1,284	70	285
2016	1,596	122	582
2017	2,813	127	713
2018	3,946	55	804
Total	9,882	393	2,336

a *Includes combinations with ELISA– or not specified, and IPT– or not specified*.

b *Includes the combinations ELISA+and IPT+, –, or not specified, and ELISA– or not specified but IPT+*.

c *Includes the combinations PCR– or not specified, ELISA+ and IPT+, –, or not specified, and PCR– or not specified, ELISA– or not specified and IPT+*.

**Figure 2:**
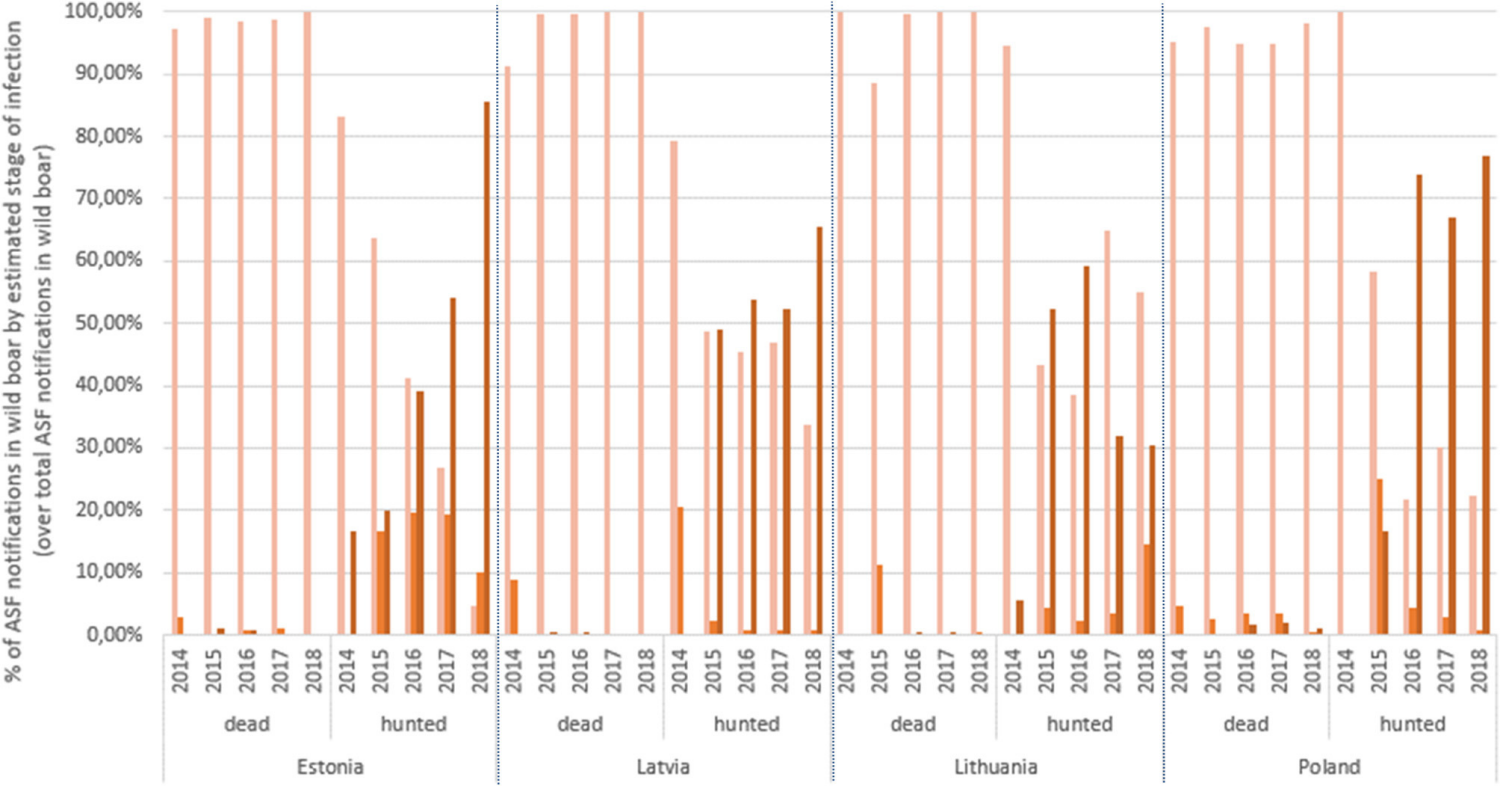
Proportion of ASF wild boar notifications by estimated stage of infection over total ASF annual wild boar notifications.

The apparent increase in notifications in Stage 3 can be better observed in Figure 2, where the proportion of notifications in each stage over the total ASF notifications in wild boar per year has been stratified by dead/hunted and by country. In dead wild boar, the predominant diagnostic result is obtained by PCR. In fact there are very few notifications (*n* = 15) in Stage 3 in dead wild boar: 2 from Latvia (one in 2015, the other in 2016) and the rest from Bialski, in Poland, in 2018. In hunted wild boar, there are some differences by country but not statistically significant according to a factorial ANOVA test. In general, the % of notifications based only on PCR results (Stage 1) has decreased since 2014 to give rise to the notifications based on antibody detection (Stage 3). A factorial ANOVA test for hunted wild boar in Stage 3 showed statistically significant differences by year, particularly from 2016 onwards (Tukey's honest significant test, confidence level = 0.99). Only Lithuania has not increased the % of antibody notifications by year. In hunted wild boar, 1,218 Stage 3 notifications are truly PCR negative, antibody positive and in 1117 PCR is not mentioned, but they are antibody positive. Out of the 1,218, 1,106 are from Latvia and exhibit an annual increasing trend (2015 = 158; 2016 = 282; 2017 = 297; 2018 = 369), 4 are from Lithuania (2015), and the remaining 108 are from Poland (2016 =1; 2017 =19; 2018 = 88). A Poisson regression model on the apparent annual increase of notifications in Stage 3 in Latvia indicates that it is statistically significant (*p* <0.01). The correlation test indicated an overall strong positive statistical association between ASF serology notifications and TWI by administrative unit (rho = 0.77, *p* = 2.2 ×10^−16^). Although still statistically significant, the correlation was weaker when considering the proportion of notifications based on antibody detection (rho = 0.35, *p* = 2.2 ×10^−4^).

**Figure 3:**
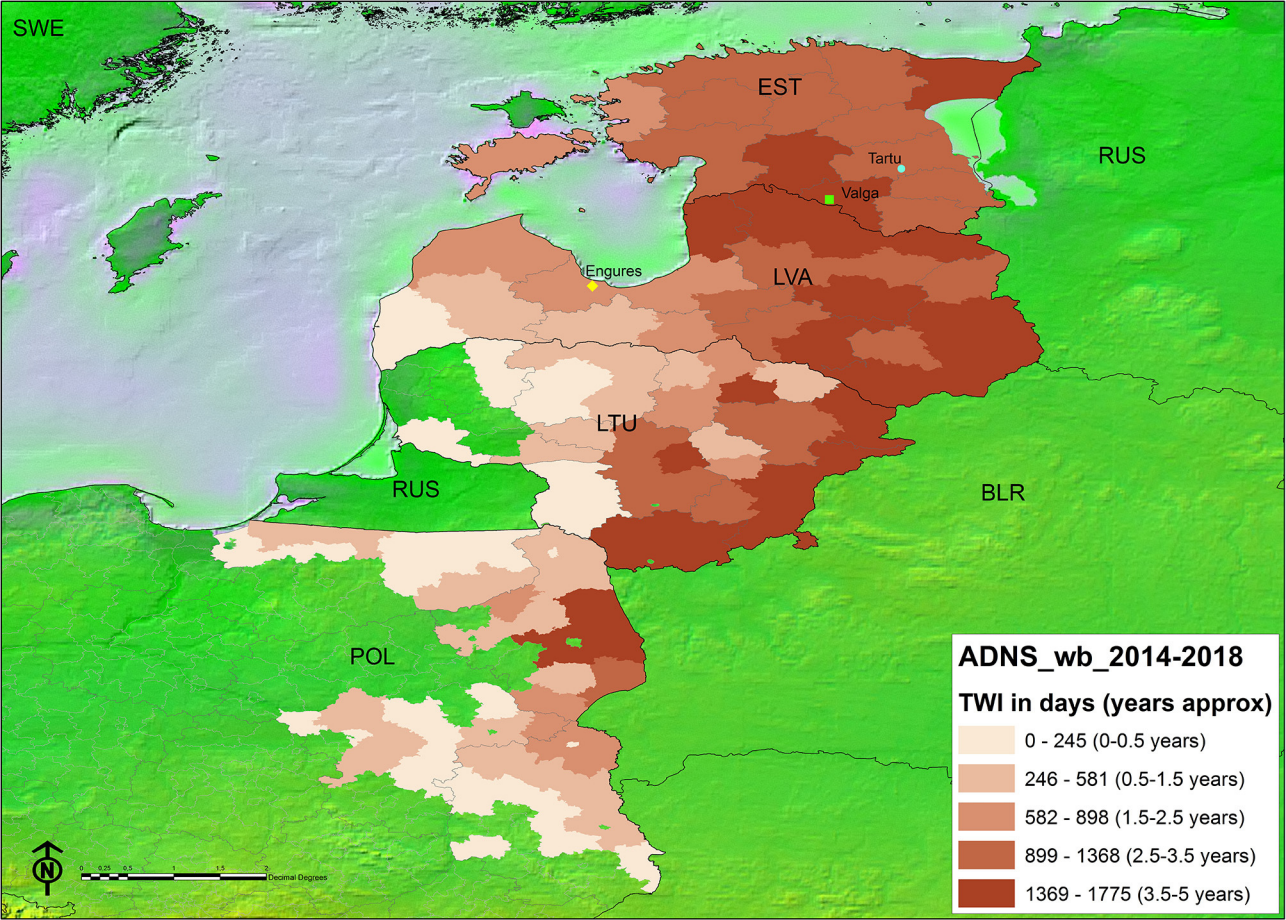
Map of the time with infection (TWI) distribution (natural breaks) by administrative unit (Data source: EU Animal Disease Notification System). Country acronyms as per ISO 3166-1 alfa-3). Points indicate the location of ASF virus of attenuated virulence characterized at the EU Reference Laboratory for ASF, from top to bottom: circle: ES15/WB/Tartu14 (5); square: ES15/WB/Valga6 (5); diamond: LV17/WB/Rie1 (6).

**Figure 4:**
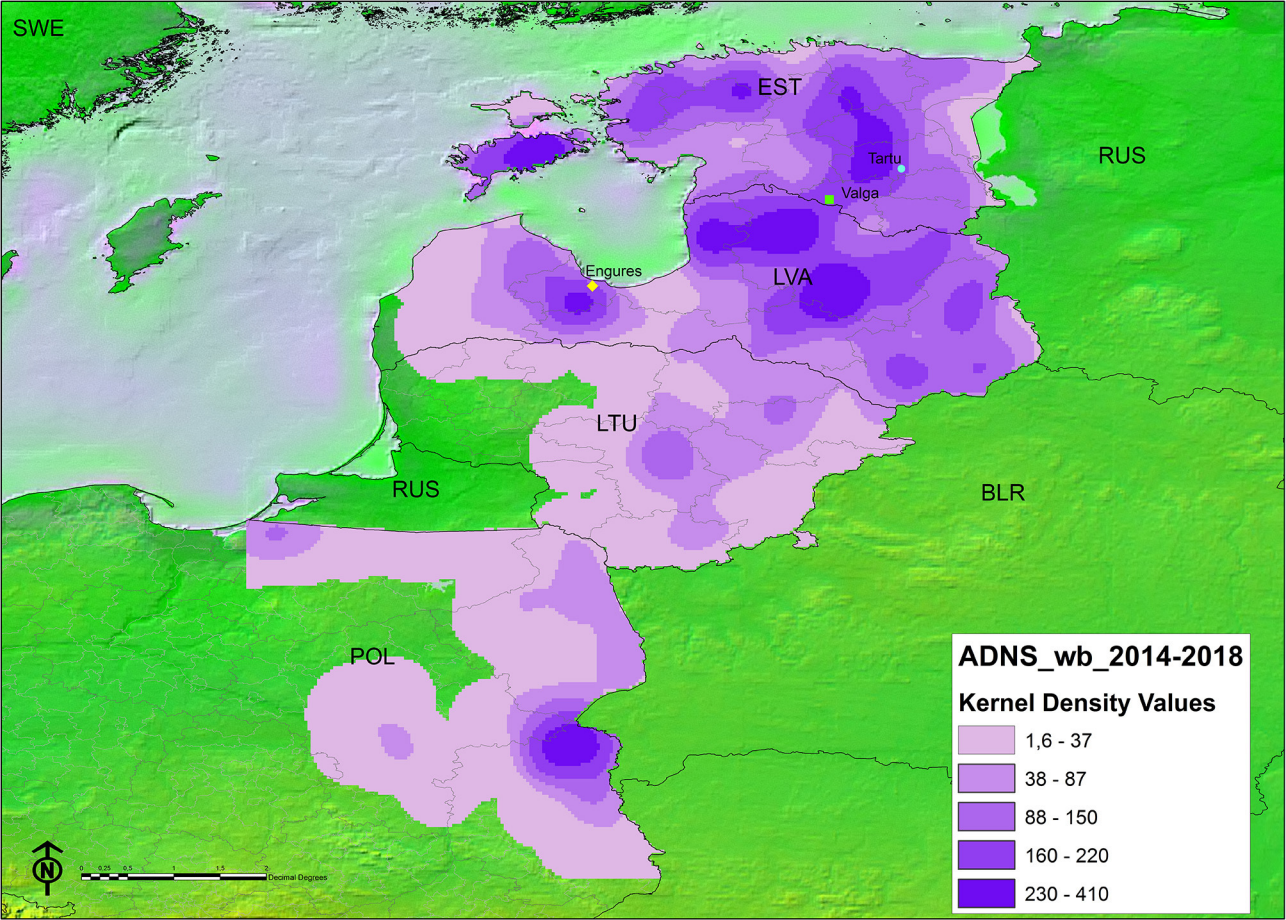
Kernel density map of antibody-based ASF notifications in wild boar classified by natural breaks (darker color indicates higher density). Points indicate the location of ASF virus of attenuated virulence characterized at the EURL, from top to bottom: circle: ES15/WB/Tartu14 (5); square: ES15/WB/Valga6 (5); diamond: LV17/WB/Rie1 (6).

The spatial representation of the TWI in each administrative unit per country can be found in Figure 3. The areas bordering Belarus and those between Latvia and Estonia, have had ASF for longer. The kernel density map of notifications in either Stage 2 or Stage 3 (Figure 4) showed that in some instances, a higher density of antibody positive notifications is present in those areas where ASF has been present for longer, particularly in Latvia. The density of antibody positive notifications is very low in the border of Lithuania with Belarus, where ASF has been present for more than 3 years. In contrast, in certain areas relatively recent in their acquisition of the infection, like the Estonian island of Saarema or the southwestern notifications in Poland, there is a higher density of serological notifications. We have represented in both maps the location of the virus that were characterized as moderately virulent by the EURL (5, 6). Both Estonian virus were isolated from 2015 outbreaks, one in Valga and the other in Tartu. In both regions ASFV has continued to circulate since 2015, since Valga is classified in the longest TWI category (3.5–5 years) and Tartu in the second longest (2.5–3.5 years). Both fall in an area corresponding to the second highest seroprevalence density category. In Latvia, the virus recovered from a 2017 outbreak in Engures was non-hemadsorbing (non-HAD). This area has a TWI of only 1.5–2.5 years, however it also falls into an area with the second highest seroprevalence density category.

## Discussion

The analyses of the evolution of wild boar ASF notifications to the European Union (EU) surveillance database (ADNS) in Estonia, Latvia, Lithuania and Poland, the four countries which have had ASF since its introduction in the EU in 2014, reveal a progressive and statistically significant increase in the percentage of notifications based on antibody positive results with either negative or assumed negative PCR result in the period 2014–2018 in hunted wild boar (Stage 3), even if the number of notifications in hunted wild boar has remained relatively stable and much lower than notifications of wild boar found dead across the whole period. The annual increase in “truly” Stage 3 (PCR negative, antibody positive) notifications was tested only for Latvia since it was the only country with consistent data across the study period.

For the analyses of ASF wild boar evolution with ADNS data, we have had to make certain assumptions. We cannot control the way the data was recorded into the system, and consequently any bias derived from data collection or entry will be accumulated. While the EU Regulation and the ADNS system ensures certain harmonization, our analysis was based mainly on classifications made from the “free text” and thus subject to our interpretation and assumptions as explained in the Materials and Methods section. For example, there is a notification in Estonia in February 2015 with 10 hunted wild boar in which ASF was confirmed by ELISA and immunoblotting. There is no information on PCR results so it has been classified as Stage 3. If correctly classified, one could interpret that as soon as that early in the epidemic there were potential survivors. Latvia also confirmed 6 hunted wild boar PCR negative and ELISA positive in a single notification. Further analyses could be performed if, in addition, information on the antibody titers were included with these type of notifications, reducing the potential bias derived from our classification method. Similarly, we are assuming that the animals classified in Stage 3 could be potential survivors. They remain potential since with the information provided it is impossible to estimate the uncertainty regarding their status. The dynamics of ASFV, widely studied in the scientific literature, show that antibodies are detectable from 1 week onwards after infection, peaking between days 10–20 post infection and then maintained at high levels over time if the animal survives (10, 11). Gallardo et al. (11) also summarize in their article that viremia has experimentally been detected by PCR as early as 3 days post-infection (dpi) in acute infections and at an average of 8.5 ± 3.6 dpi in subacute infections. Also, that in pigs surviving acute or subacute infections, viral DNA has been detected in blood for up to 78 days, but with several peaks, similarly to the excretion pattern.

ASFV has circulated for almost 12 years in Eastern Europe, of which the last nearly 5 years correspond to spread in the EU (mainly in wild boar). The probability of co-circulation of virus with different virulent degrees is higher than at the beginning of the epidemic, as is the probability of prolonged “high risk periods” (time between infection and field detection) that would allow a “silent” spread of infection. The “high risk period” was estimated to be between 7 and 20 days in domestic pig farms in Estonia between 2014 and 2017, where all antibody positive animals were also PCR positive (25). In wild boar, since there has been up to now an active component of surveillance for hunted boars in infected areas, this offered an opportunity to evaluate the likelihood of ASFV spread by “healthy” animals. In terms of laboratory results, more antibody positive and PCR negative field samples are to be expected if the surveillance design still contemplates hunting to test wild boar for control and eradication purposes at least. This is because antibodies for ASF are assumed to remain for life, but viremia, when it persists, it is with intermittent peaks and therefore easier to miss under surveillance conditions.

The representation of the time with infection (TWI) per administrative unit is a quick and easy way to capture the evolution of ASF spread, particularly when prevalence data cannot be measured adequately because of a changing and often imperfect denominator data. The wild boar population density in the affected areas has changed over the last years probably due to ASF deaths and to the application of drastic depopulation measures to fight ASF (26). Other analytical studies on surveillance data have used wild boar density estimates dividing the hunting records per year by the sum of the hunting grounds [(10) for Estonia] or by reconstructing a numerical value per map cell based on habitat suitability maps combined with abundance data based on hunting records [(27) for Poland]. For our study, we preferred to use the TWI since our primary interest was to analyze surveillance results assuming that, in the light of ASFV dynamics, an increase in time of PCR negative and antibody positive results could reflect a higher probability of animals surviving the infection. However, if the population of wild boar has indeed decreased, rising percentages of notified seropositive animals would also be expected naturally if the number of animals surviving the infection remained constant in time. So far the survival rate is not known. Similarly, if further field observations reveal that there is a difference in incidence between age groups as was evidenced with classical swine fever (28), the interpretation of TWI and seropositive findings should also take this difference into account. There are higher TWI values along the Belarusian border with EU countries. Belarus was infected before the EU (in 2013) and, together with Russia, it was the suspected origin of wild boar notifications in the EU (29). The TWI also indicates the direction of spread, east to west, since in the latter ASF has appeared later. Finally, the TWI also allows further epidemiological investigation into areas in which the infection could be perpetuating. In this sense, we would expect to find more ASF potential survivor wild boar, particularly if the virus circulating in those areas correspond to strains of attenuated virulence. A potential increase in the number of animals surviving the infection could also reflect a balance in the host-virus interaction either because of a possible attenuation of ASFV virulence, a higher immunity of the host or a change in the routes of transmission of ASFV that lead to lower viral infection doses. The evidence of circulation of attenuated strains is scarce for the moment and is mainly restricted to experimental observations. The experimentally identified attenuated virus (5, 6) were obtained from areas in which ASF has been present as soon as 1.5–2.5 years with the infection. All three identified ASFV attenuated strains correspond to areas with a high density of antibody positive notifications. It is hard to expect that these strains be identified in the field if the surveillance design does not contemplate their potential detection. So far, the EU legislation (Council Directive 2002/60/EC) intends a 100% sampling of the whole hunting bag in restricted areas, applying an optimal strategy for their identification. In Latvia, results have showed an annual increasing trend of potential survivors: 1,106 notifications of hunted animals in Stage 3 were PCR negative and antibody positive (2015 = 158; 2016 = 282; 2017 = 297; 2018 = 369). This fact strengthens the importance of enhancing testing of shot wild boar for surveillance purposes because, opposite to what Schulz et al. (10) stated, the probability of finding more animals surviving the infection should not be considered a rare event in the current epidemiological situation. Finding dead wild boar can be hard, particularly if there are only a few hours of light like is the case in the Baltic countries in winter. In addition, it can also be difficult to find dead animals under harsh weather conditions, like snow or rain. Wild boar surveillance data is imperfect by nature and its epidemiological interpretation is of utmost importance to understand the extent of the infection in the field.

The main area in which a high TWI does not correspond with a high density of antibody positive notifications is in the Lithuanian border with Belarus. Lithuania and Poland have fewer wild boar notifications from hunted animals than Estonia and Latvia. Assuming a similar surveillance effort for hunted wild boar across countries, one could interpret that Poland and Lithuania are experiencing more recent and acute infections, while in Estonia and Latvia more moderately pathogenic forms could have started to occur. Lithuania experienced outbreaks in commercial hunting grounds densely populated during 2017, and from 2016 there was also a compensation scheme to notify wild boar found dead (30). Both aspects could explain the increase in ASF wild boar found dead. In Estonia, there has been an increase in the proportion of antibody positive notifications, and the latest area to be infected, the island of Saarema, concentrates a high number of notifications with serology. Nonetheless, overall, there is a strong statistical correlation between the number of notifications with an antibody positive result and TWI per administrative unit, which is what was expected given the common regulatory framework that harmonizes surveillance efforts among countries.

In addition to the current information provided by countries in the official notifications, it would be extremely useful to include the quantitative result of the antibody titration. Antibody titration allows to estimate the time since infection, which would provide further insight on the epidemiological situation by allowing to identify recovered and asymptomatically infected animals. The most commonly test used for ASF antibody detection is the ELISA but it is only suitable for serum or plasma (31). ASF antibodies persist for many months and even years (20, 32) and serological assays are the most efficient way, due to their simplicity and relatively low cost to detect animals with unspecific signs of disease due to infection with moderately virulent strains (11). For antibody detection in blood, exudate tissues or body fluids, IPT is the test of choice (11). IPT has a higher sensitivity than the ELISA and is used as a confirmatory test for ELISA positive sera from ASF free areas or when doubtful ELISA results are obtained from endemic areas or serum samples are poorly preserved (31). However, IPT requires specific expertise and training to interpret the results and they are not commercially available. For this reason, the availability of a commercial confirmatory serological assay has been identified as a priority for the near future (11). The continuous presence of ASF in certain areas together with the never-ending threat of reintroduction from endemic areas or with a tendency to become endemic should be considered to update the surveillance and control plans.

In conclusion, the TWI provides a relatively fast and easy tool to assess the evolution of ASFV infection by geographical area even with limited population data. Surveillance based on ASFV detection by PCR and serology is a powerful source of data to assess the status of the epidemic in wild boar despite its imperfect nature, and allows to follow up the evolution of further potential survivors.

## Data Availability Statement

Data were from the EU Animal Disease Notification System database. The authors don't have permission to share the datasets. Requests to access these datasets should be directed to Marta Martínez-Avilés, marta.sanidadanimal.info@gmail.com.

## Author Contributions

All authors contributed to the design, analyses, interpretation of results, and writing of the manuscript.

### Conflict of Interest

The authors declare that the research was conducted in the absence of any commercial or financial relationships that could be construed as a potential conflict of interest.
