# Corrigendum: Evolution of the ASF Infection Stage in Wild Boar Within the EU (2014–2018)

**DOI:** 10.3389/fvets.2020.581766

**Published:** 2020-10-26

**Authors:** Marta Martínez-Avilés, Irene Iglesias, Ana De La Torre

**Affiliations:** Centro de Investigación en Sanidad Animal (CISA), INIA, Valdeolmos, Spain

**Keywords:** antibodies, epidemiology, surveillance, moderately virulent virus, survivor, African Swine fever, wild boar

In the original article, there was a mistake in Table 1 as published. **In the third column of the table, first row, under Stage 2 it should be stated in brackets (PCR+, AB+)**^**b**^. The corrected Table 1 appears below.

**Table 1:** Annual distribution of ASF notifications in wild boar by diagnostic test/s used and estimated stage of infection.

**Year**	**Stage 1**	**Stage 2**	**Stage 3**
	**(PCR+, AB-)^a^**	**(PCR+, AB+)^b^**	**(PCR–, AB+)^c^**
2014	243	19	2
2015	1,284	70	285
2016	1,596	122	582
2017	2,813	127	713
2018	3,946	55	804
Total	9,882	393	2,336

a *Includes combinations with ELISA– or not specified, and IPT– or not specified*.

b *Includes the combinations ELISA+and IPT+, –, or not specified, and ELISA– or not specified but IPT+*.

c *Includes the combinations PCR– or not specified, ELISA+ and IPT+, –, or not specified, and PCR– or not specified, ELISA– or not specified and IPT+*.

The authors apologize for this error and state that this does not change the scientific conclusions of the article in any way. The original article has been updated.

